# Cervicogenic headache treated by acupuncture based on *jin* theory: study protocol for a randomized controlled trial

**DOI:** 10.1186/s13063-019-3478-1

**Published:** 2019-07-10

**Authors:** Youkang Dong, Taipin Guo, Lei Xu, Chunlin Wang, Guanfen Wen, Zhiyong Zhao, Lianhai Duan, Mei Zou, Yong Xiang, Shu Wang

**Affiliations:** 1 The First Affiliated Hospital of Yunnan University of Chinese Medicine (Yunnan Provincial hospital of Traditional Chinese Medicine), Kunming, 650021 China; 2 Tianjin University of Traditional Chinese Medicine, Tianjin, 300193 China; 3 School of Acupuncture-Tuina and Rehabilitation, Yunnan University of Traditional Chinese Medicine, Kunming, 650500 China; 4 The First Affiliated Hospital of Tianjin University of Traditional Chinese Medicine, Tianjin, 300193 China; 5 The First Affiliated Hospital of Bengbu Medical College, Bengbu, 233000 Anhui China

**Keywords:** Acupuncture, Cervicogenic headache, Randomized controlled trial, Study protocol

## Abstract

**Background:**

Numerous randomized trials involving acupuncture treatment for cervicogenic headache (CEH) have been conducted in recent years, but the evidence for its effectiveness is not clear. Hence, we designed a randomized trial to evaluate the efficacy and advantages of acupuncture for treating CEH.

**Design:**

This is a parallel-design, two-arm, patient-assessor blinded, randomized, sham-controlled clinical trial. A total of 166 patients with CEH aged from 18 to 70 will be recruited and assigned randomly into a *jin* acupuncture group and a pseudo acupuncture group at a 1:1 ratio; they will receive 12 sessions of real acupuncture and sham acupuncture for 4 weeks, respectively, during the study. The primary outcomes are pain degree (PD) and pain rate (PR) calculated by the PainVision analyzer, as well as parameters detected by surface electromyography (SEMG). The secondary outcomes will be measured with the short-form McGill Pain Questionnaire (SF-MPQ), range of motion (ROM) of the neck, the Northwick Park Neck Pain Questionnaire (NPQ), the 36-item short-form Health Survey (SF-36), the Self-Rating Anxiety Scale (SAS), and the Self-Rating Depression Scale (SDS). Clinical assessments will be evaluated at baseline and in the fourth week as well as in the eighth and sixteenth weeks. Adverse events will be noted and recorded for the safety evaluation.

**Discussion:**

This study will provide high-quality evidence of the value of acupuncture based on *jin* theory for treating CEH.

**Trial registration:**

Chinese Clinical Trial Registry, ChiCTR1800015316. Registered on 22 March 2018. Updated version AMCTR-IOR-18000157. Registered on 1 April 2018.

**Electronic supplementary material:**

The online version of this article (10.1186/s13063-019-3478-1) contains supplementary material, which is available to authorized users.

## Background

Cervicogenic headache (CEH) refers to a group of syndromes characterized by unilateral preponderance of headache, restricted movement of the neck, and hypersensitivity of the occipital neck region caused by degeneration of the cervical vertebra and detriment of the cervical soft tissue [1]. An epidemiological investigation showed that approximately 2.5% of individuals suffer from CEH, with an age distribution between 30 and 50 years and a ratio of men to women of 1:4 [2]. The International Headache Society (IHS) considers CEH as a special disorder different from common types of headache [3], with published and revised reports in 2004 and 2013, respectively [4, 5]. Although the pathological mechanism of CEH is still disputed, with causes focused mainly on musculoskeletal disorders in the cervical spine [6] or convergence afferent signal disturbance of the trigeminal nerve and the upper cervical nerves [7], there are various interventions being performed according to the accumulated experience, varying knowledge scopes, and professional trends of doctors, such as drugs (including narcotic drugs) [8], nerve block [9], pulse radiofrequency [10], Western medicine [11], acupuncture [12], Tuina [13], and Chinese medicine [14].

As a vital constituent of traditional Chinese medicine (TCM), acupuncture is well known and used in some countries. It has played a very important role in dealing with diseases associated with cervical spondylosis in China [15]. Some clinical trials demonstrated acupuncture to be superior to conventional methods regarding curative effects and unexpected events in the treatment of CEH [16, 17]. *Jin* theory, as an important branch of meridian theory, was demonstrated to be effective in the treatment of pain [18]. Clinically, according to TCM, *jin* disease basically appears as aches, clonuses, spasms, rigidity, relaxation, and limb weakness, symptoms which are very similar to the manifestation of muscle, tendon, myofascial membrane, ligament, and neuropathic lesions. Therefore, from the perspective of functional anatomy, *jin* refers to a complex system combining local anatomical morphology and significant characteristics mainly involving muscles, tendons, myofascial membranes, ligaments, and nerves, etc. [19]. CEH is a kind of *jin* disease, and its pathogenesis is closely related to the *pain point of jin* formed by pathological changes in muscles (cervical extensor muscles, myofascial fascia, and tendons) and nerves (occipitalis major nerve and occipitalis minor nerve) and abnormality of proprioception [19, 20]. The aponeurosis attaches to the articular surface or vertebrae and becomes a stress concentration point due to the great stress caused by keeping the head and neck in a low flexion position. Long-term stress concentration can lead to the occurrence of energy metabolism crises of the muscle fibers, fibrosis of muscles, and other lesions, which contribute to form the tension zones and sensitive points of muscle fibers (*gather* and *knot* in TCM), inducing pain and distal referred pain when pressed [21]. In addition, when tension and spasm occur in the muscle where the nerve passes through, compression or stimulation symptoms are prone to be produced at the outlet of the nerve [22]. Therefore, taking the outlet of the nerve as an acupoint to perform acupuncture should show positive significance for the relief of symptoms of CEH.

At present, more attention is paid to the evaluation of headache, ignoring symptoms and signs of the neck and occipital region. Also, abundant non-acknowledged therapies have been adopted conventionally in control groups, which has weakened the clinical study quality of acupuncture for CEH. In this study, we focus on the main and secondary symptoms and signs involving headache as well as pain and stiffness of neck-occipital region, and we aim to verify the value of acupuncture based on *jin* theory. The study and its final conclusion will provide a reasonable and high-quality clinical trial contributing to the popularization and application of acupuncture for CEH.

## Methods and design

### Study design

**Fig. 1:**
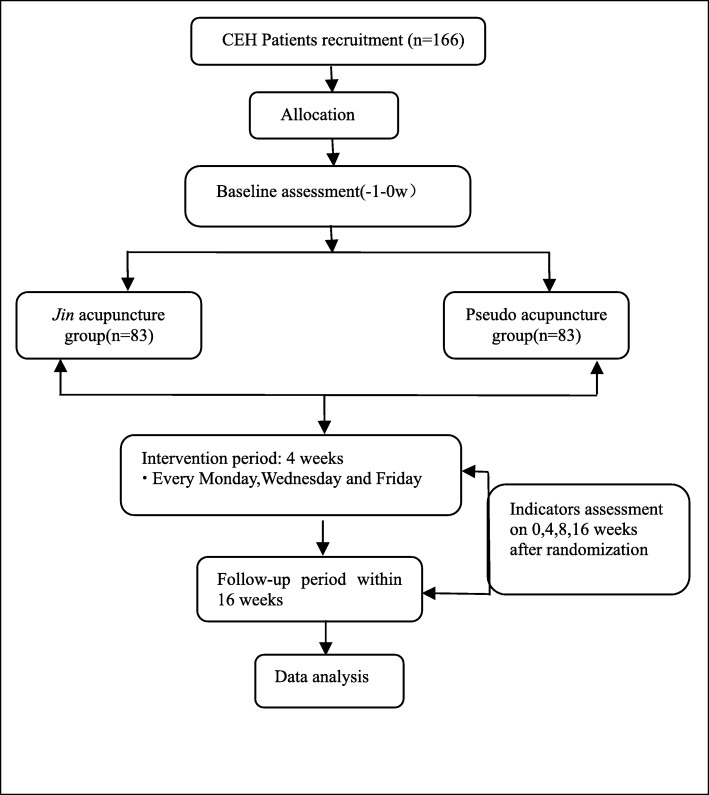
Flowchart of this trial

This is a two-arm, parallel-design, patient-assessor blinded, clinical randomized controlled trial (RCT) aiming to evaluate the differences of efficacy between acupuncture based on *jin* theory and sham acupuncture for CEH. A total of 166 patients will be recruited from the Department of Acupuncture-Tuina and Rehabilitation of the first affiliated hospital of Yunnan University of Traditional Chinese Medicine. Recruitment advertisements will be placed on the network and recruitment posters. Eligible participants in each group will have an equal chance of being allocated randomly to obtain real or sham acupuncture treatment. The primary outcomes are objective parameters detected by devices (PainVision analyzer and surface electromyography [SEMG]); the secondary outcomes are assessed by scales. This study will last 16 weeks including 1 week for baseline, 4 weeks for treatment, and 12 weeks for follow-up. Methods and data from this study will contribute to the feasibility and simplicity of acupuncture for CEH patients. This protocol was registered on 22 March 2018 (ChiCTR1800015316, AMCTR-IOR-18000157). The updated protocol was changed on 26 April 2019. Figure 1 shows a flowchart of the study. Additional file 1 provides the Standard Protocol Items: Recommendations for Interventional Trials (SPIRIT) checklist; Additional file 2 shows the Standards for Reporting Interventions in Controlled Trials of Acupuncture (STRICTA) checklist.

### Participants

#### Study population and sample size

Patients with CEH meeting the diagnosis of the International Classification of Headache Disorders, 3rd edition (ICHD-3) formulated by the IHS [5] will be enrolled. The reduction of the visual analog scale (VAS) average score was 6.37 to 2.31 after triple acupuncture treatment compared to 6.37 to 2.52 for conventional acupuncture treatment according to a relevant study [23]. We propose to set the VAS of the short-form McGill Pain Questionnaire (SF-MPQ) average score as 2 and 4 for the *jin* acupuncture group and pseudo acupuncture group, respectively. Given a significance level of 0.05, a standard deviation of 5, and power of test of 0.95, and considering a 10% loss rate, 166 participants in total are needed with 83 in each group.

#### Inclusion criteria

Volunteers who meet the following criteria modified from the Headache Classification Subcommittee of the IHS will be recruited: (1) headache originating from the neck and perceived in one or more regions of the head and/or face; (2) clinical, laboratory, and/or imaging evidence of a disorder or lesion within the cervical spine or soft tissue of the neck known to be a valid cause of headache; (3) clinical signs implicate a source of pain in the neck, and the headache can be attributed to the neck disorder or lesion; (4) aged from 18 to 70 years, both males and females; (5) have not received any treatment within 2 weeks; (6) willing to abide by and sign informed consent.

#### Exclusion criteria

Volunteers who meet any of the following criteria will be excluded: (1) headache related to tumor, cervical vertebral tuberculosis, spinal canal occupying lesions, or scoliosis or resulting from intracranial infection, cerebroma, subarachnoid hemorrhage, or other diseases; (2) have had operation for cervicogenic headache; (3) serious underlying diseases of important organs such as heart, liver, kidney, brain, or blood vessels, hematopoietic disorders or diabetes; (4) pregnant or lactating women; (5) unable to understand or record scoring indicators; (6) serious mental disorder, anxiety, or depression; (7) have received other treatment or involved in other clinical trials.

#### Dropout criteria

Patients who are unable to comply with this study and fail to achieve the expected efficacy, or who experience severe changes in their condition during treatment, will be dropped from the study.

### Randomization, allocation concealment, and blinding

The eligible patients who meet the criteria will be randomly distributed to the *jin* acupuncture group and the pseudo acupuncture group with an allocation proportion of 1:1. A statistician who is not taking part in the clinical intervention will use SPSS 25.0 (IBM, Chicago, IL, USA) to generate a random allocation sequence. All of the random code information will be stored in sealed opaque envelopes by a well-trained specified assistant, who will randomize and inform the acupuncturists of the treatment assignments by phone. The allocation concealment procedure will not be exposed until the clinical trial is finished completely. Assessments and measurements of the participants will be carried out before and after the treatments and also at the follow-ups by different individuals of the research team. Researcher A will be responsible for the baseline assessment, administration of the questionnaire, and the measurement of cervical range of motion. Researcher B will be in charge of detecting the SEMG signal on the cervical extensor muscles. Researcher C will detect and record the pain degree (PD) and pain rate (PR) with the PainVision analyzer. Researcher D will be responsible for the analysis of SEMG data. A licensed acupuncturist who has worked for more than 18 years will perform acupuncture interventions on the patients, and patients will be treated in a shielded clinic room and deprived from knowing something about the pattern of treatment.

### Interventions

**Fig. 2:**
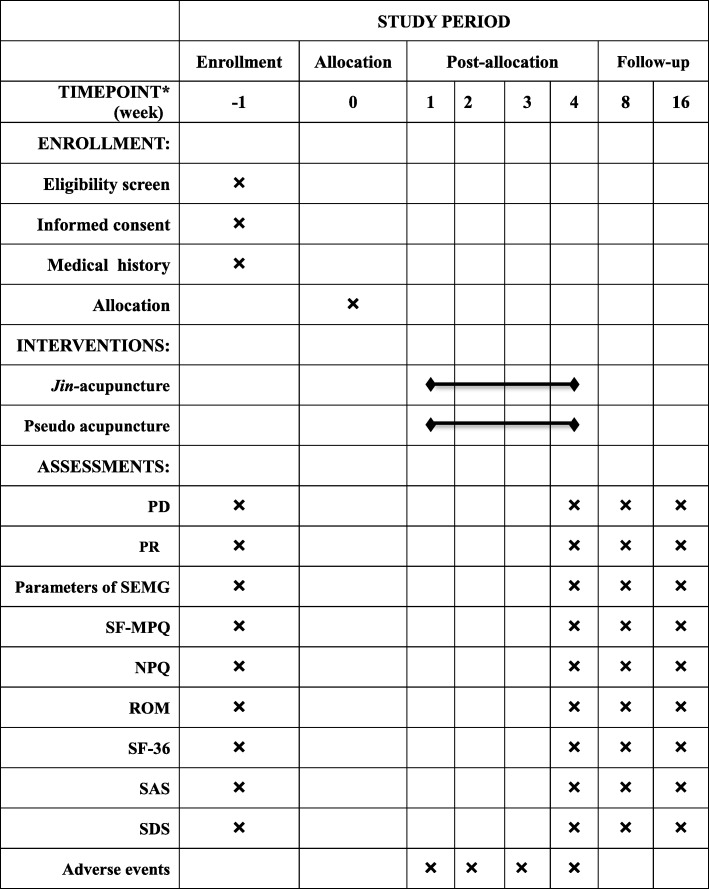
SPIRIT figure adopts PD and PR of PainVision analyzer, parameters of surface Electromyography (SEMG), short-form McGill Pain Questionnaire (SF-MPQ), range of motion (ROM) of the neck, Northwick Park Neck Pain Questionnaire (NPQ), Self-Rating Anxiety Scale (SAS), Self-Rating Depression Scale (SDS) for enrollment, interventions, and assessments

Patients in the *jin* acupuncture group will receive 12 sessions of acupuncture during a period of 4 weeks, implemented by finding the *gather* and *knot* of positive reaction, the points of both stress concentration and neurogenic stimulation in cervical extensor muscles according to TCM *jin* theory and modern biomechanical principles of soft tissue. Sterile and disposable filiform needles (Suzhou Acupuncture & Moxibustion Appliance Co. Ltd. Jiangsu, China) 40 mm in length and 0.30 mm in diameter will be inserted in accordance with the permissible depth and angle of the affected side acupoints until the *de qi* sensation is achieved. Meanwhile, acupuncture techniques of lifting-thrusting and twirling-rotating will be implemented to facilitate the arrival of *qi* involving a sensation of aching, numbness, heaviness, or distension around the acupoints. The needles will be retained for 25 min and stimulated every 8 min intermittently, with each acupoint being stimulated for 10 s. Participants in the pseudo acupuncture group will receive the sham acupuncture using filiform needles on points 5 cm apart away from the acupoints used in the *jin* acupuncture group. The skin will be punctured without any manipulation and stimulation for 25 min to avoid the *de qi* sensation, with the same sessions and courses as the *jin* acupuncture group. The baseline period is 0 to week 1 with one evaluation time before participating. The course of treatment and observation period will be three sessions a week totaling 12 sessions during the course. After 4 weeks of treatment, we will provide one evaluation session. The follow-up evaluation will be performed in the eighth and sixteenth weeks. The study schedule is shown in Fig. 2; the treatment details for each group are listed in Table 1.

**Table 1:** Treatment details for each group

Group	Acupoint/location (affected side)	Manipulation
*Jin* acupuncture	I. Stress concentration points a. In the midpoint of the connection between BL9 and BL10 b. In the midpoint of the connection between GB20 and SJ17 II. Positive reaction points c. GB8 d. In the midpoint of temporomandibular joint III. Nerve stimulation points e. The sensitive point of occipital protuberance of upper line of posterior occipital region f. Sensitive points on the posterior border of the upper third of the sternocleidomastoid muscle g. Sensitive spots in intertubercular sulcus of the second transverse process	a, b, f, g and GB34: Perpendicular insertion with 1.2 *cun* c: Oblique insertion with 0.8 *cun* toward the ipsilateral body d, e: Oblique insertion with 0.5 *cun* down Retaining needles for 25 min Lifting-thrusting, twirling, and rotating for *de qi* sensation
	Matching point: GB34	
Pseudo acupuncture	5 *cm* away from a, b, c, d, e, f, and g; 8 points in total	Retaining needles for 25 min only, pricking skin with no manipulation to avoid *de qi* sensation

Number of needle insertions per subject per session will be 8

### Outcome measures

#### Primary outcome measures

The following primary outcome measures will be used to evaluate the effectiveness at weeks 4, 8, and 16 after randomization, respectively, compared with the baseline.

1. Pain degree (PD) and pain rate (PR): With a measurement accuracy of 0.1 μA, 0.3 ms pulse width, and 50 Hz pulse frequency, both indicators will be calculated using the current perception threshold (CPT) and pain equivalent current (PEC) with the PainVision analyzer according to the following formula [24, 25]:
  $$ PD=\frac{PEC- CPT}{CPT}\times 100\kern0.5em PR=\frac{PEC}{CPT} $$
2. Surface electromyography (SEMG): With the patient in a prone position, a sticker will be placed on the affected side cervical extensor muscles (2 cm lateral to the posterior midline of the neck). The SEMG signal will be detected through an eight-channel module ME6000 biomonitor (Mega Electronics Ltd., Kuopio, Finland) as the operator successively applies 25% (maximum voluntary contraction, MCV), 50% MCV, and 100% MCV resistance, respectively, to the posterior occipital area. The system will automatically record and transmit the signal to generate the original image, and indicators of muscle fatigue including median frequency (MF), mean power frequency (MPF), average rectified value (ARV), and root mean square (RMS) will be calculated by Mega Win software for statistical analysis [26].

#### Secondary outcomes

The secondary outcomes are measured as described below.

1. Short-form McGill Pain Questionnaire (SF-MPQ): This questionnaire comprises Pain Rating Index (PRI), Present Pain Intensity (PPI), and VAS items. The PRI includes sensory and affective parts with negative, mild, moderate, and severe pain (from 0 to 3) representing the degree, while the PPI values from 0 to 5 represent different degrees of pain. A VAS number of 0 represents no pain, and 10 represents unbearable pain [27].
2. Northwick Park Neck Pain Questionnaire (NPQ): The NPQ has been commonly used in clinical research and has shown high validity and practicability for neck pain assessment [28]. It consists of nine items involving the degree and duration of pain, numbness of upper extremities, sleep, and social activities as well as other quality of life characteristics. The total score is 100, and high scores mean severe disability from neck pain.
3. Range of Motion (ROM) of the neck: This reliable and valid tool, consisting of two gravity goniometers and a compass goniometer, is used to assess the degree of cervical spine movement involving the cervical movement distance of bilateral rotation and flexion without undergoing pain [29]. This measurement is performed by a specific senior researcher, and the mean values will be recorded to facilitate the analysis.
4. Short-form 36-item Health Survey (SF-36): The health-related quality of life and correlation factors including physical function and daily functioning are evaluated by a 5-point scale [30].
5. Self-Rating Anxiety Scale (SAS) and Self-Rating Depression Scale (SDS): The SAS is a relatively simple clinical tool to analyze the subjective symptoms of patients. It is applicable to adults with anxiety symptoms and has a wide range of applications. The SDS can fairly directly reflect the subjective feelings of patients with depression and their changes in treatment. It is mainly applicable to adults with depressive symptoms. Compared to the SDS, the SAS can better reflect the subjective feelings of patients with anxiety tendencies [31].

### Data collecting and monitoring

Data will be recorded in electronic case report forms (eCRFs) by specific outcome assessors, and will be established and monitored by the Data Monitoring Committee (DMC) of the First Affiliated Hospital of Yunnan University of Chinese Medicine. Inspectors will inspect the data, check the study protocol compliance and nonscheduled informed consent documents, and evaluate the conditions of participant recruitment and data quality of this trial. During the evaluation process, the researchers, including acupuncturists and statisticians, will have no eligibility to see the data. Any amendments to this study protocol should be tracked and dated for submittal of a new version to the committee.

According to the data-sharing policy of the Chinese Clinical Trial Registry (CHICTR), data will be transmitted to the official website of Yunnan University of Chinese Medicine (www.ynutcm.edu.cn) within 6 months after this trial is completed. Data will be opened on the condition of publication of the main study findings. In order to protect the participants’ confidentiality, all of the external investigators should be asked to sign the agreement. Any adverse events (AEs) or accidents will be observed, reported, and monitored in time until they are resolved. The safety of the trial will be assessed before and after the treatment to avoid AEs, and a strict investigation and relative follow-up monitoring will be performed if they occur.

### Adverse events

Any possible AEs related to acupuncture intervention that occur, including nausea, fainting, subcutaneous hemorrhage, local infection, stuck needles, or needle breakage will be checked and treated. Details of AEs will be recorded in the CRF by the acupuncturist and security administrator. Severe AEs will be reported to the Ethics Committee and DMC, and compensation will be considered if necessary. Patients who are unwilling to persist with the treatment will be removed from the trial.

### Statistical analysis

All of the data will be entered in the CRFs and input into the database by two independent researchers, calculated by means of the SPSS 25.0 software package. Data analysis and comparison will be performed based on the intention-to-treat (ITT) population and the per protocol (PP) population, contributing to the integrity and objectivity of this study. The ITT population consists of all participants who have been randomized and received at least one session of acupuncture after the baseline. PP refers to the patients who have completed the study and did not violate the protocol. A two-sided value of *P* < 0.05 will be defined as the statistical significance, categorical variables in two groups will be analyzed by chi-squared or Fisher’s exact test, normally distributed data will be analyzed by Student’s *t* test, and non-normally distributed data by the Mann-Whitney *U* test. Continuous variables in two groups including primary outcomes (the PainVision and SEMG parameters) and secondary outcomes will be compared using a general linear model with repeated measurement methods at all time points.

## Discussion

CEH is a subtype of headache derived from a lesion of the cervical spine with accompanying detriment of the surrounding soft tissues [32]. Evidence has indicated that the effective rate and VAS of acupuncture for CEH is obvious compared to non-steroidal anti-inflammatories and other treatment methods, and few adverse reactions occurred [33–36]. Given that most studies paid more attention to the assessment of the headache, ignoring symptoms and signs of the surrounding soft tissues as well as ROM of the cervical spine [12, 37], the quality and reliability of the studies still needs to be improved by carrying out well-designed clinical trials.

From the perspective of *jin* theory of TCM and modern soft tissue biomechanics [38, 39], this study proposes to treat CEH with specific points: the stress concentration point and positive reaction point. Both of the points are related to trigger pain points and tenderness points, which contain attachment points of occipital-cervical *jin* to the bones. The points of *gather* and *knot* of *jin* are formed due to detriment of greater stress from fascia and tendons to the bones [40]. Thus, loosening anomalous *jin* can alleviate pain and tension in local soft tissues [41]. In addition, the necessity of therapeutic effect of the nerve stimulation point which is an outlet point of superficial branches of nerves in CEH is also taken into consideration [42]. Pain is likely to be induced by prolonged flexion of the head and neck and may also be caused by cold stimulation [43].

Studies have shown that superficial punctures under the skin have an analgesic effect on fibromyalgia and other types of chronic pain [44–46]. This trial aims to adopt sham acupuncture, the international general method, as the control condition to evaluate the curative effectiveness of acupuncture based on *jin* theory. With the use of CEH-related indicator assessment, strict quality control, the guidance of professional experts, and an 8–16 weeks follow-up period, this trial will provide strong evidence for the use of acupuncture treatment for patients with CEH.

### Trial status

This trial (study protocol version pro l, dated 2018-01-16) is ongoing. The recruitment began on 2 April 2018, and the approximate completion date of the trial is 30 April 2020.

## Additional file

Additional file 1: SPIRIT 2013 checklist. (DOC 134 kb)

Additional file 2: STRICTA checklist. (DOCX 24 kb)

## Data Availability

Not applicable.

## Abbreviations

AE: Adverse event
CEH: Cervicogenic headache
CRF: Case report form
ICHD-3: International Classification of Headache Disorders, 3rd edition
MCV: Maximum voluntary contraction
MF: Median frequency
MPF: Mean power frequency
NPQ: Northwick Park Neck Pain Questionnaire
PPI: Present Pain Intensity
PRI: Pain Rating Index
RCT: Randomized controlled trial
ROM: Range of motion (of the neck)
SAS: Self-Rating Anxiety Scale
SDS: Self-Rating Depression Scale
SEMG: Surface electromyography
SF-MPQ: Short-form McGill Pain Questionnaire
TCM: Traditional Chinese medicine
VAS: Visual analog scale
